# Cutaneous adaptive immunity and uraemia: a narrative review

**DOI:** 10.3389/fimmu.2024.1464338

**Published:** 2024-09-27

**Authors:** Noushin Zibandeh, Zehua Li, Graham Ogg, Matthew J. Bottomley

**Affiliations:** ^1^ Chinese Academy of Medical Sciences Oxford Institute, University of Oxford, Oxford, United Kingdom; ^2^ Department of Dermatology, Oxford University Hospitals NHS Foundation Trust, Oxford, United Kingdom; ^3^ MRC Translational Immune Discovery Unit , University of Oxford, Oxford, United Kingdom; ^4^ Oxford Kidney and Transplant Unit, Oxford University Hospitals NHS Foundation Trust, Oxford, United Kingdom

**Keywords:** adaptive immune, cutaneous changes, immunosenescence, inflammation, uraemia

## Abstract

Chronic kidney disease affects 1 in 10 people globally, with a prevalence twenty times that of cancer. A subset of individuals will progress to end-stage renal disease (ESRD) where renal replacement therapy is required to maintain health. Cutaneous disease, including xerosis and pruritus, are endemic amongst patients with ESRD. In the uraemia-associated immune deficiency of ESRD, impaired circulating immune responses contribute to increased infection risk and poorer vaccination response. Clinical manifestations of dysregulated adaptive immunity within the skin have been well-described and have been posited to play a role in cutaneous features of ESRD. However, our understanding of the mechanisms by which adaptive immunity within the skin is affected by uraemia is relatively limited. We provide an overview of how the cutaneous adaptive immune system is impacted both directly and indirectly by uraemia, highlighting that much work has been extrapolated from the circulating immune system and often has not been directly evaluated in the skin compartment. We identify knowledge gaps which may be addressed by future research. Ultimately, greater understanding of these pathways may facilitate novel therapeutic approaches to ameliorate widespread cutaneous symptomatology in ESRD.

## Introduction

Chronic kidney disease (CKD) is a major cause of non-communicable morbidity and mortality, with approximately 850 million cases worldwide (10% of the global population) (1). This represents a twenty-fold greater prevalence compared to cancer (2). By 2040, CKD will be the fifth leading cause of years of life lost (3). A subset of individuals progress to end-stage renal disease (ESRD), requiring renal replacement therapy or kidney transplantation.

Advanced CKD and its associated physiological milieu (uraemia) has been long linked with systemic immune dysfunction. Reduced delayed-type hypersensitivity (DTH) responses and dampened responses to vaccination indicates this extends to peripheral sites such as skin (4, 5). Uraemic immune dysfunction culminates in increased infection risk and poorer outcomes (6, 7).

Skin issues are widespread in patients with advanced CKD (8). Shared pathogenic mechanisms may cause both renal disease and induce dermatopathology [for example, systemic lupus erythematosus or Henoch-Schönlein Purpura (IgA vasculitis)]; exploration of these is beyond the scope of this review. Independent of CKD aetiology, many cutaneous symptoms are overrepresented in uraemic patients. For example, pruritus affects at least half of patients, with increasing prevalence as disease progresses (9–12). Other uraemic skin manifestations include xerosis, acquired ichthyosis, altered pigmentation, purpura, and nail/mucosal changes (8). Cutaneous manifestations in CKD have been recently reviewed (13). Immunopathology has been postulated to contribute to many of these conditions.

As a barrier organ, almost every cell type in the skin is capable of initiating and maintaining an immune response. Previous experimental methods for studying the contribution of each to cutaneous immunity were limited in that they could analyze only a small number of markers simultaneously, necessitating pre-determined hypotheses, and/or lacked the ability to perform dynamic, functional assessments of behaviour.

New approaches, such as single-cell and spatial transcriptomic profiling, offer the ability to examine cell behaviour and signalling pathways across thousands of genes, often with the benefit of single-cell resolution or preservation of spatial context (14). These have revealed novel perspectives on cellular interactions in both skin homeostasis and pathology (15), uncovering previously unappreciated heterogeneity within cutaneous populations and permitting new insights into immune-stroma-epithelial crosstalk (16), permitting deployment of targeted therapies such as biologics targeting signalling pathways into clinical practice (17). Cutaneous immune profiling has revealed spatially and transcriptomically distinct leucocyte subpopulations, allowing distinction of specialised, skin-resident subsets, those transiently circulating through tissue, and those recruited in inflammation (18). Taken together, a picture is emerging of an incredibly complex immune network within the skin that acts in concert to protect against pathogens.

Development of adaptive immune responses within the skin require successful coordination of a series of communication networks, often commencing with Langerhans cell (LC) or dermal dendritic cell antigen acquisition and migration to draining lymph nodes (enhanced by inflammatory signalling from keratinocytes, fibroblasts and melanocytes) and culminating in recruitment of circulating lymphocytes through chemokine and cytokine signalling and upregulation of adhesion molecules by endothelial cells (19–23). Resolution of inflammation is accompanied by local retention of a small number of tissue-resident lymphocytes which subsequently act as sentinels and first responders, promoting rapid cutaneous memory responses following antigen re-encounter (24, 25). Cellular immune responses within skin are supported and complemented by humoral responses, with constitutive local production of antibody which is upregulated in the setting of inflammation (26–28). These processes are modulated by regulatory cutaneous leucocyte populations, such as regulatory T cells, which dampen inflammation (29). Dysregulation in any one of these steps may lead to skin pathology.

Much remains unclear about the interaction between uraemia and immune dysfunction, and how this extends to the skin. This review therefore presents an overview of the field, identifying knowledge gaps where new techniques may identify novel pathways and therapeutic approaches to ameliorate disease.

## Changes in circulating adaptive immunity with uraemia

Kidney failure correlates with accumulation of circulating pro-inflammatory markers, an exhausted T cell phenotype, and skewing of T follicular helper subtypes, typically becoming more marked as kidney impairment progresses to ESRD (30, 31). Increased prevalence of virus-associated cancers, tuberculosis, and impaired vaccination response to T cell-dependent antigens in ESRD patients indicate a demonstrable impact of T cell impairment (32).

Circulating lymphopenia is common, becoming most profound in ESRD (33–35). T-cell lymphopenia may also be driven by increased T cell susceptibility to apoptosis (36). The mechanisms responsible for the impaired responses of CD4+ T lymphocytes in dialysis patients remains unclear. However, disruptions in T lymphocyte activation may be caused by the presence of uremic toxins and proinflammatory cytokines or may be associated with early proliferative senescence (37).

Advanced CKD may lead to imbalanced T cell responses, with a shift towards Th2 polarisation (38, 39). Dialysis may impair T cells’ ability to produce Th1 cytokines upon *ex vivo* stimulation, potentially indicating ‘exhaustion’ (40). Accordingly, ESRD is associated with accumulation of circulating T cells with an exhausted phenotype (41). Exhaustion, arising as a result of chronic antigenic stimulation and particularly described in the setting of cancer and chronic viral infection, leads to dampened effector function and the upregulation of inhibitory receptors (42, 43). Exhausted T cells may revert to fully functional T cells under certain conditions, but ultimately leads to cell death and impaired immune response if conditions persist (42–44). Other polarisation fates also may be affected, with a shift in the Th17: Treg axis towards Th17 responses described in CKD (45–47).

Despite a shift towards Th2 polarisation, usually associated with antibody-mediated responses, the production of antigen-specific effector memory CD4+ T cells after vaccination is severely impaired in patients with ESRD (32). This is crucial for achieving an adequate humoral response. As CKD progresses in adults, there is a reduction in circulating B cell number, particularly driven by loss of immature B cells and accumulation of double-negative B cells, which may play an inhibitory role in humoral responses (48, 49). Elevated plasma levels of IL-7 are found in uraemia, which promote the transformation of pre-B cells into B cells (50). B cell lymphopenia appears to be relevant prognostically in ESRD, with lower circulating B cell count associated with elevated all-cause mortality risk (51).

Children with CKD exhibit notably reduced numbers of memory type B cells, despite normal transitional B cell counts (52). This may be due to increased B cell susceptibility to apoptosis in uraemia, linked to reduced Bcl-2 expression (53). This suggests that B cell deficiency may be multifactorial with enhanced susceptibility of uraemic B cells to apoptosis and impaired maturation in the periphery through resistance to differentiation and survival signals.

## Alterations in cutaneous immunity in uraemia

Uraemic immune dysfunction has been particularly studied in circulating populations, whereas understanding the effect upon peripheral immunity, particularly the skin, is limited. However, reduced delayed type hypersensitivity response and increased skin allograft survival in patients with advanced CKD (54), both of which require T cell mediated immunity, support the concept that immune dysfunction extends to the skin in uraemia. Most research in this area has described morphological rather than functional shifts in cutaneous immunity, based on histological analysis rather than dynamic evaluation.

Priming of cutaneous adaptive immune responses are likely to be altered in uraemia. Patients with advanced CKD demonstrate reduced LC density within the epidermis (55, 56). Whether this represents enhanced turnover, loss of self-renewal or altered migration remains unclear. LCs are equipped with Toll-like receptors (TLR) that allow them to directly detect pathogen-associated molecular patterns (PAMPs) from viruses and bacteria (57). This capability facilitates their phenotypic maturation and differential cytokine production, highlighting the crucial role of LCs in regulating skin immune responses. Intrarenal TLR2 and TLR4-mediated signalling play a role in initiating and aggravating CKD in various settings (58); extrarenal signalling similarly mediated through uraemic toxins may also be enhanced (59). Identifying the key endogenous ligands for TLRs involved in renal disease will be crucial for developing TLR blockade as a potential therapeutic approach for these conditions.

Keratinocytes also express pattern recognition receptors, including TLR that recognise highly conserved structures including microbial-derived lipopolysaccharides, flagellins and DNA sequences (60–63). Activation via TLR leads to secretion of proinflammatory cytokines and chemokines, driving recruitment and activation of circulating leucocytes, and upregulation of antigen presenting capability (64–67). Uraemic toxins stimulate TLR directly *in vitro* (68), and therefore may act *in vivo* to drive inflammation and trigger chronic LC migration out of the skin.

**Figure 1:**
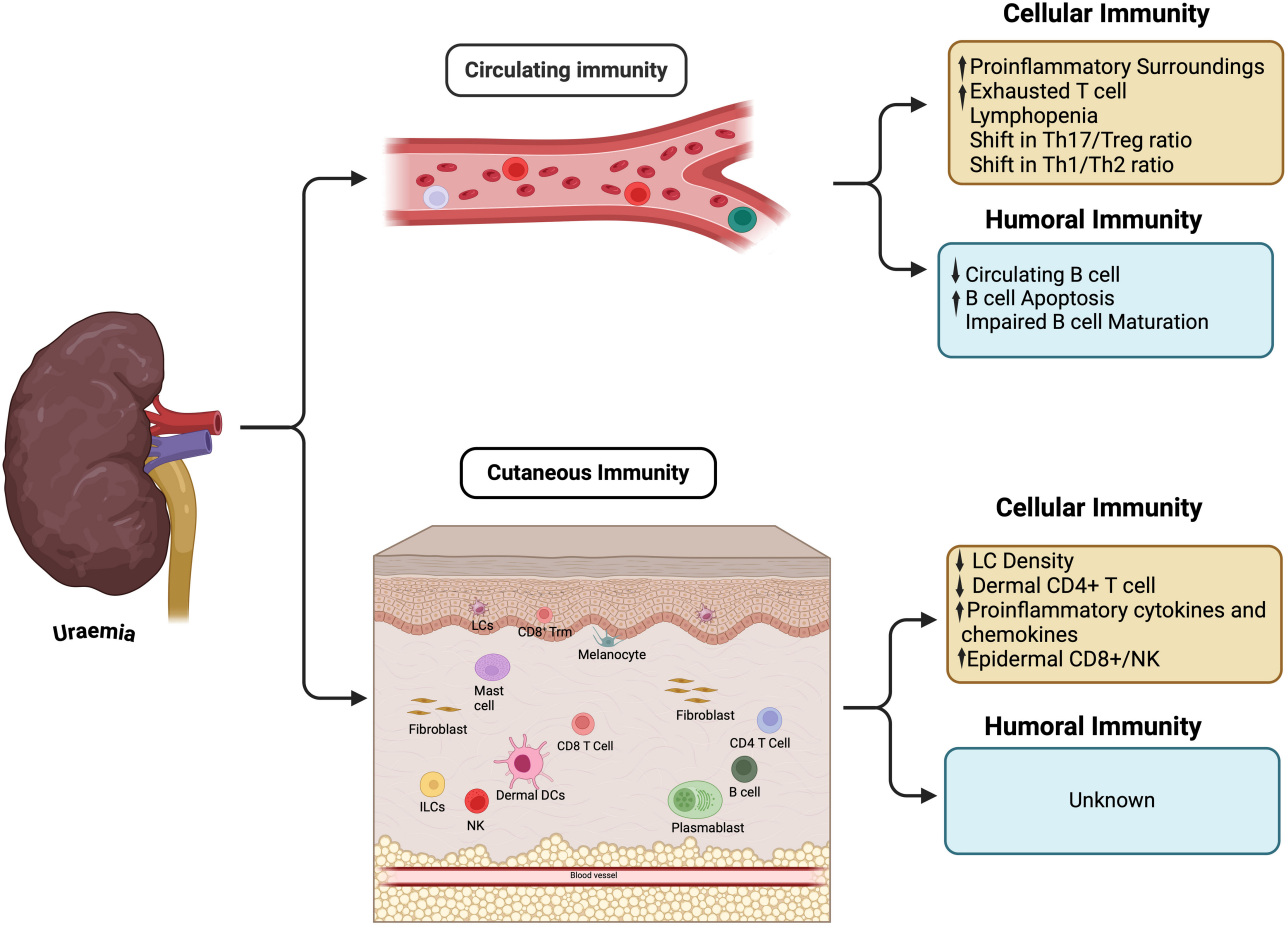
Immunity changes in circulation and cutaneous tissue. ILCs, Innate lymphoid cells; LCs, Langerhans cells; NK, Natural killer; Trm, Tissue resident memory.

Fibroblasts, present in the dermal layer, are increasingly being recognised as active players in cutaneous immunity. Heterogeneity in fibroblast behaviour is increasingly apparent leading to delineation of subsets which modulate cutaneous immunity in health and disease (69–72). Indirect evidence, such as impaired response to growth hormone signalling (73), and impaired wound healing (74), suggests uraemia impacts cutaneous fibroblast behaviour, however the effect upon modulation of immune response remains unexplored. Shifts in both the type and distribution of T cell populations have been described in skin from non-diabetic patients with advanced CKD, with increased epidermal CD8+/NK and reduced dermal CD4+T cell density compared to non-uraemic controls (56). Changes in T cell localisation and density support the clinical observation that priming, and recruitment of the cutaneous adaptive immune response is altered in CKD. Though uraemia clearly impacts circulating B cells, it is not known whether this reflects changes in the cutaneous compartment. Alterations in circulatory and cutaneous immunity previously described in the setting of advanced CKD are summarized in **Figure 1**.

## Drivers of uraemic immune dysregulation

### Changes in the microenvironment may drive uraemia-associated immune dysfunction both directly and indirectly

#### Direct effect: microinflammation and uraemia-associated toxicity

Uraemia is associated with chronic, low-level systemic inflammation termed ‘microinflammation’ (75), This is driven by oxidative stress associated with accumulation of proinflammatory cytokines, advanced glycation end-products (AGE), metalloproteases and overproduction of reactive oxygen species (76–79). Renal replacement therapy (i.e. dialysis) artificially removes many of these toxins from the body albeit in a relatively inefficient manner (80); this means that many of these products continue to accumulate even with treatment. Dialysis, particularly haemodialysis, may contribute to immune dysfunction and inflammation through dialysis membrane-driven activation of complement cascades and stimulation of circulating granulocytes, monocytes and T cells, and has been recently reviewed (81).

Microinflammation may directly influence cutaneous immunity and indeed uraemic pruritus may represent a clinical manifestation of this altered neuro-immunological interaction, though mechanistic understanding is limited (82, 83). Up to 40% of patients report this symptom, with pruritic individuals exhibiting increased serum C-reactive protein, as well as dysregulated circulating Th1/Th2 lymphocyte balance, compared to non-pruritic controls. Serum C-reactive protein and IL-6 levels are increased in pruritic uraemic individuals, with circulating T cells demonstrating Th1 skewing, compared to non-pruritic controls (84). There is an increase in the cytokines produced by Th1 lymphocytes including IFN-γ, IL-6, and TNF-α (76). In contrast, levels of IL-31, a Th2 lymphocyte pruritogenic cytokine, are increased in uraemic individuals (85). Microinflammation may impact upon cutaneous immunity through other mechanisms. For example, acute inflammatory signalling leads to upregulation of inflammatory cytokines by local fibroblasts followed by resolution of inflammation. However, in the setting of chronic inflammation fibroblast behaviour changes in a heterogenous manner - some differentiate into myofibroblasts leading to extracellular matrix deposition, which stiffens tissue and compromises leucocyte infiltration (86). Others differentiate into inflammatory fibroblasts that promote tissue retention of leucocytes. Which pathway is dominant in uraemic skin inflammation is unknown, but can be surmised to the former given the reduction in cutaneous leucocyte number in CKD.

Microinflammation may underpin the loss of LC seen in uraemic skin, though promotion of LC migration to draining lymph nodes (19). In the setting of chronic microinflammation unchecked LC migration may deplete the normally self-renewing cutaneous LC pool, with altered transendothelial trafficking preventing replenishment from the circulation (87).

Over 100 uraemia-associated toxic metabolites have been described, many of which have not been evaluated for their immunological effect (88). Indoxyl sulfate (IS) and p-cresyl sulfate (PCS) are two well-studied solutes, which exemplify how uraemic toxins may directly influence skin immunity and drive inflammation (89).

*In vitro* and animal models indicate IS and PCS may induce keratinocyte and fibroblast inflammation and drive histamine-independent itch (90–93). IS may also act directly upon skin-resident leucocytes, leading to differentiation and activation of proinflammatory macrophages (94–97). Ligation of the aryl-hydrocarbon receptor (AhR) on monocytes and macrophages by IS was shown to induce secretion of inflammatory mediators, driving chemotaxis and recruitment of effector T cells that induce endothelial apoptosis and promote further inflammation (98, 99). PCS induces circulating monocyte activation but impairs antigen processing (100). A recent study demonstrated PCS and IS synergise in contributing to the increasing proportion of pro-inflammatory, intermediate monocytes in CKD patients (101), suggesting that the cocktail of uraemic solutes present *in vivo* may have greater effects upon immunity than the study of individual molecules *in vitro*.

Uraemia-associated toxic metabolites may modulate T and B cell behaviour directly. IS inhibits Th2 cell differentiation, leading to loss of IL-4 producing CD4+ T cells, in an AhR-dependent manner in a murine model of asthma (102). This could drive Th1 skewing, inflammation and skin pathology in uraemic individuals. Blood PCS concentration correlates with levels of terminally differentiated CD8+ T cells in ESRD patients, potentially suggesting a mechanistic link between uraemic toxins and premature immune aging in patients with ESRD (33). B cells may be similarly activated by uraemic toxins through AhR. PCS directly leads to B cell lymphopenia in animal models (103), whilst AhR co-stimulatory signalling plays a critical role in driving B cell proliferation, but also regulates class switching and plasma cell differentiation, promoting development of a regulatory phenotype (104, 105).

Uraemia-associated microinflammation and toxin accumulation result in vascular inflammation, leading to endothelial dysfunction, prothrombotic changes, increased vascular permeability and accelerated vascular ageing (106, 107). *In vitro* studies have shown that the rupture of cell-cell junctions and reduced transendothelial electrical resistance indicated by uraemic toxins lead to structural damage of the endothelial monolayer, which could be associated with vascular injury and the development of chronic vascular diseases (108–110). This may enhance uraemic toxin and circulating inflammatory cytokine diffusion into peripheral tissues, including the skin (111). It’s unclear how this alters adhesion and tissue migration dynamics of circulating leucocytes, but conceivably could lead to impaired tissue infiltration if endothelial behaviour and signalling is dysregulated.

Ageing is associated with antigen-independent inflammation, leading to cutaneous recruitment of monocytes. Active vitamin D3 has been demonstrated to reduce this inflammation (112). 25-Vitamin D3 undergoes 1α hydroxylation in the kidney in order to acquire biological activity - consequently many patients with advanced CKD demonstrate functional vitamin D3 deficiency, requiring use of active vitamin D3 to prevent mineral bone disease. 25-Vitamin D3 reverses uraemia-associated inflammation in monocytes *in vitro* (113), but whether uraemia-associated skin inflammation may respond to this therapy in a clinical setting remains to be seen.

Electrolyte disturbance, associated with impaired clearance by the kidneys, may directly contribute to immune dysfunction. Total body sodium is frequently increased in uraemia. Skin is a major storage organ for sodium, where it exerts pleiotropic effects (114). Sodium may inhibit M2 macrophage differentiation and regulatory T cell function (115, 116), whilst simultaneously activating T cells (117). Similarly, hyperkalaemia is a common finding in patients with advanced CKD, with elevated potassium concentration having been shown to inhibit CD8+ T cell effector function (118). To what extent either of these mechanisms are active within uraemic skin are unclear but may play a contributory role to cutaneous immune dysfunction.

AGE are covalent compounds created by spontaneous (enzyme-independent) reaction of long-lived proteins, lipids and other macromolecules with monosaccharides and amino acids (119). Over time, this leads to pathophysiological cross-linked structures which alter tissue dynamics, including in skin (79). AGE accumulation is accelerated in the presence of oxidative stress, such as that found in CKD. Their accumulation in uraemia can be clinically quantified by fluorescence assessment (120) and may associate with poorer clinical outcomes (121, 122). AGE may act as a trigger for innate immune activation through TLR and a receptor for AGE (RAGE) expressed on cutaneous myofibrovascular populations, dendritic cells and monocytes (123–125), whilst potentiating oxidative stress and microinflammation in a positive feedback loop (126). Conversely, enhanced local fibrosis and tissue rigidity resulting from AGE accumulation has been suggested to prevent chemotaxis, particularly in neutrophils (127).

Taken together, uraemic patients accumulate electrolytes and AGEs due to reduced renal clearance and oxidative stress. Electrolyte imbalances may cause immune dysfunction by directly inhibiting the adaptive immune system, whereas AGEs may act more via initiation of innate immunity, exacerbating microinflammation. The latter synergises with deficiency in functional vitamin D3, leading to antigen-independent inflammation.

#### Indirect effect: uraemia-driven immunosenescence

A bidirectional link between chronic kidney disease and ageing is increasingly apparent. Whilst chronological age is a major risk factor for chronic kidney disease (128), progression of biological ageing may itself be accelerated in the uraemic milieu and is not reversed following kidney transplantation (129).

The immune system undergoes a progressive change in composition and functional capacity during biological ageing, termed immunosenescence. This manifests as increased susceptibility to infectious diseases, reduced response to vaccination, and elevated mortality risk in the general population (130–132). An underlying feature is ‘inflamm-ageing’, a chronic low-level non-specific inflammatory state with increased serum levels of C-reactive protein, IL-6 and IL-1β (133).

Although immunosenescence affects the entire immune system, most research has focused on the adaptive immune response, particularly in circulating T cells. Hallmarks of immunosenescence include thymic involution, a reduced naïve/memory ratio in circulating T and B cells and progressive loss in the functional and replicative capacity of lymphocytes (134–136).

T cell immunosenescence is typified by the accumulation of terminally differentiated, effector cells, characterized by loss of costimulatory molecules such as CD27 and CD28 and expression of killer cell lectin-like receptor subfamily G (KLRG-1) and CD57 (137, 138). Functionally, these cells demonstrate decreased replicative ability, increased production of proinflammatory cytokines and impaired TCR signal transduction with increased reliance upon killer receptor signalling (139–141).

The circulating immune landscape in uraemia shares many similarities with immunosenescence (142, 143). An average difference of 20 years between the immunological and chronological age in uraemic patients has been observed, indicating a prematurely aged T cell compartment (144). Microinflammation and oxidative stress drives accelerated thymic involution and premature loss of naïve T cell output (145). Accelerated peripheral T cell turnover may further contribute, with uraemic T cells demonstrating premature alterations in age-associated changes in TCR signalling cascades (146), and expression of early apoptotic markers (147, 148), suggesting increased susceptibility to activation-induced apoptosis (34). Many uraemic/ageing-related changes seen in circulating blood are also present in lymph nodes, albeit without accumulation of terminally differentiated T cells (149).

Cytomegalovirus (CMV) plays a major role in driving circulating T cell immunosenescence (150, 151). Uraemic patients demonstrate more frequent CMV reactivation, potentially creating a cycle of progressive immune impairment and increased CMV turnover (152, 153). The recent discovery of CMV-driven immune responses against senescent fibroblasts in skin by CD4+ T cells raise the question of whether a parallel CMV infection-immune circuit dominates cutaneous aging/uraemia (154).

Optimal responses against antigens by T cells are achieved through a broad TCR repertoire. However, reduced/skewed circulating TCR repertoire diversity is a hallmark of both immunosenescence and uraemia, and may therefore contribute to impaired responses (155, 156). Skewing occurs as accumulated terminally differentiated T cells are oligoclonal, often targeted against CMV (157, 158).

**Figure 2:**
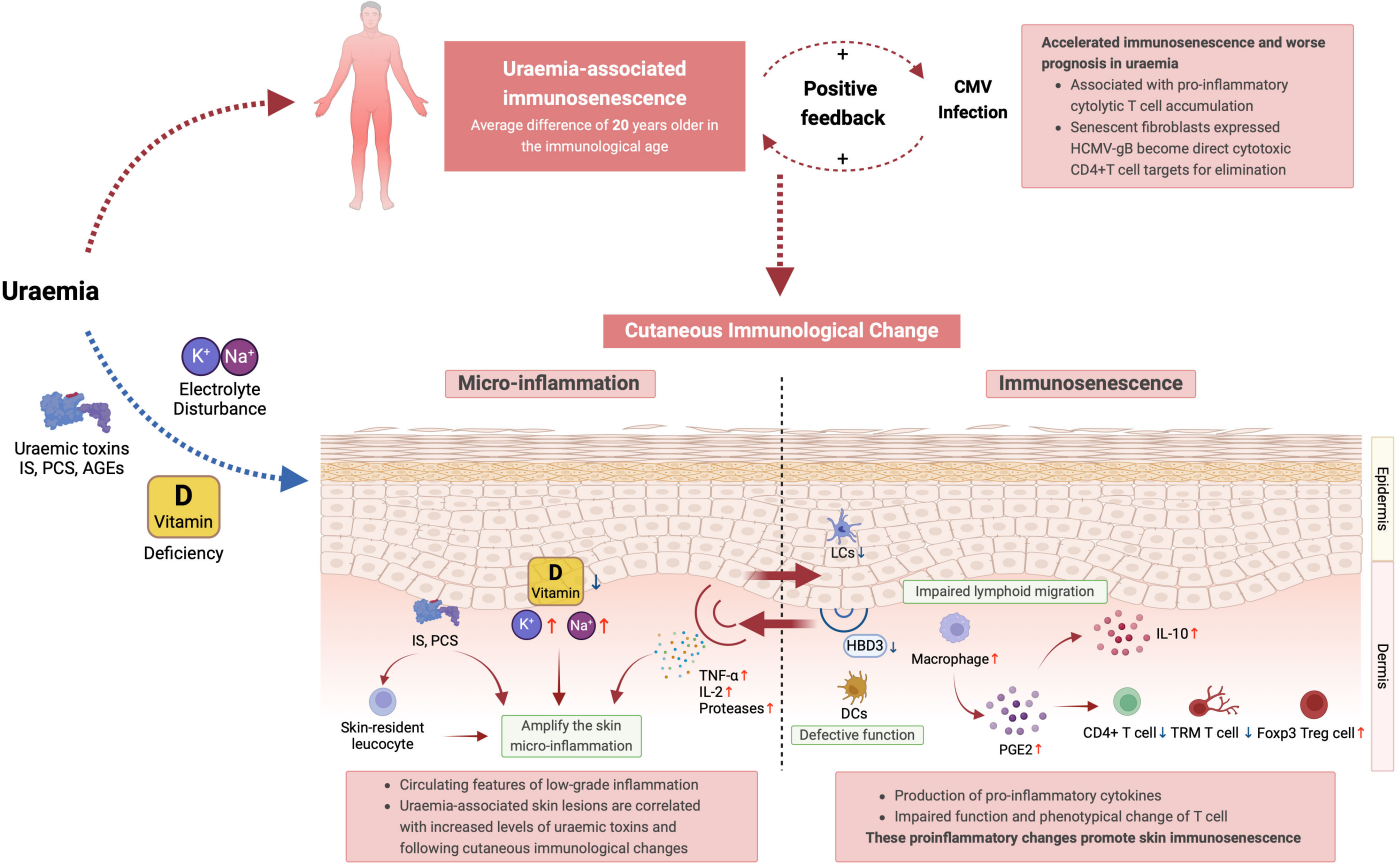
Summary of the direct and in-direct effects of uraemia upon cutaneous immunity. IS, indoxyl sulfate; PCS, p-cresyl sulfate; TCR, T cell receptor; NK Cell, natural killer cell; DCs, dendritic cells; HCMV-gB, human cytomegalovirus glycoprotein B; LCs, langerhans cells; HBD3, human beta-defensin 3; PGE2, prostaglandin E2; TRM T cells, tissue-resident memory T cells; Foxp3 Treg cells, forkhead box P3 regulatory T cells.

Other features of premature ageing, such as decreased CD4/CD8 ratio, are also seen and may be particularly marked in paediatric populations with renal impairment (35, 159, 160). Similarly, circulating B cells, NK cells, and dendritic cells demonstrate changes in ESRD comparable to aged individuals (50, 56, 161). Direct and in-direct effect of uraemia upon cutaneous immunity summarised in **Figure 2**.

##### Cutaneous immunity in immunosenescence

Given the functional and phenotypical overlap between uraemia-associated immune deficiency and immunosenescence in blood, one may hypothesise this extends to the periphery also, although this has not been previously explored. Studies of ageing in skin may therefore be informative by extrapolation to uraemia and cutaneous immunity.

Cutaneous ageing may be associated with impaired adaptive immune priming and homing. A loss of LC and impaired lymphoid migration is seen in older people, akin to the loss of LC described in uraemia (162–164). Alongside impaired lymphocyte priming, this may also reduce the epidermal pool of defensins (165). Dermal dendritic cells also demonstrate defective trafficking, signalling and T cell stimulation from aged skin (166).

Macrophage-derived signalling is a major source of chronic, antigen-independent, low-level inflammation within aged skin (167). Aged skin demonstrates an altered response to damage, leading to enhanced recruitment of prostaglandin E2 (PGE2)-secreting inflammatory monocytes (168). These monocytes inhibit effector T cell activation and proliferation and induce expansion of FOXP3+Treg, which may explain their accumulation in aged skin (169–173). Their behaviour in uraemia is unknown.

In elderly individuals, cutaneous T cells maintain their density, diversity, and cytokine production, representing a long-lived, highly stable reservoir of immunity against infection and malignancy (174, 175). Decreased responses to Varicella Zoster virus (VZV) and tumour protection in older adults have not been directly attributed to an intrinsic defect within ageing CD4 TRMs (176). Instead, enhanced inhibitory signalling upon T cells from other leucocytes, particularly myeloid populations, may create an immunoregulatory microenvironment (177). Counterintuitively, excessive non-specific production of pro-inflammatory cytokines may cause inhibition of antigen-specific T-cell function (178).

Fibroblast senescence plays a major role in contributing to age-related chronic skin inflammation. Senescent fibroblasts secrete inflammatory mediators (termed the senescence associated secretory phenotype or SASP). This drives local inflammation, both in an antigen-independent manner but also through direct targeting of senescent cutaneous fibroblasts by effector T cell populations (154). It is feasible that uraemia amplifies production or downstream effects of SASP-related inflammation and skin ageing.

Studies on blood suggest significant overlap in ageing- and uraemia- associated immune dysfunction. Limited available data do provide tentative indications that this overlap may extend to skin, though further studies are needed to evaluate the function of cutaneous T cells in uraemia and how this mirrors biological ageing.

## Outstanding questions and future research directions

The existing literature on the effect of uraemia upon immunity highlights a number of areas of limited understanding. Studies to date have typically focused on single populations, *in vitro* models or low plex, targeted gene expression or histological analyses. This has created inconsistences and disconnects.

One such example is how uraemic micro-inflammation seems to drive a Th1 shift in T cells, demonstrated by accumulation of cytolytic, senescent CD4+ and CD8+ T cells. However, the literature also suggests a shift towards a Th2 phenotype with reduced Th1 cytokine production amongst T cells. How do we reconcile this? The classical Th1/Th2 paradigm may be insufficiently complex to explain T cell behaviour in the setting of uraemia, leading to dysregulated T cell differentiation - as exemplified by impaired vaccine responses despite a Th2-skewed state. Alternatively, findings from studies in blood may not be reflective of the peripheral tissue microenvironment.

Deeper evaluation of cell behaviour in the setting of uraemia is needed to understand this more clearly. Novel profiling approaches, such as single-cell transcriptomic profiling and spatial profiling, offer the opportunity to re-examine cutaneous cell behaviour in an unbiased way, evaluating the whole transcriptome simultaneously across multiple cell populations. In addition to highlighting pathways driving intrinsic cell dysfunction, ‘stepping back’ to evaluate the relationship between spatially-approximate cells within tissue may reveal novel ligand-receptor interactions and signalling networks suitable for therapeutic manipulation. Spatial transcriptomic profiling offers the opportunity to do this using small quantities of archived skin samples without need for specific tissue processing prior to embedding (14).

Finally, it is unclear whether we can reverse this immune dysfunction. The parallels between ageing and uraemia raises the question of whether therapeutic approaches to slow or reverse tissue senescence and improve immune function may also be effective in CKD. ‘Senolytic’ agents, such as smal molecules like rapamycin (179), chimeric antigen receptor-T-cell (CAR-T) and TCR therapy (180) and immune checkpoint inhibitors (181) have shown encouraging potential in combating cellular senescence. Limiting CMV turnover could slow immune ageing - a small phase I study found improved vaccination response and reduced number of senescent CD4+ T cells in patients with vasculitis when treated with the antiviral valacyclovir (182). Interventional studies in patients with CKD are needed to evaluate whether these are effective approaches to improve uraemia-associated immune dysfunction.

## Conclusion

Uraemia is commonly associated with skin disease, with relatively few specific therapies available for uraemic dermatopathology. In the setting of advanced kidney disease, altered immunity may play a critical contributory role in driving cutaneous symptomatology and predisposing to poorer antigen-specific responses.

Our understanding of these processes, outlined in this review, indicates notable alterations in the immunity of uraemic patients, yet the precise underlying causes of many of these changes remain largely elusive and have focused on circulating rather than tissue-resident leucocyte populations. While numerous studies have described the peripheral immune response in uraemic patients, there has been limited investigation into cutaneous immunity.

Our review highlights knowledge gaps in this field and emphasises the need for mechanistic investigations to identify novel therapeutic avenues to ameliorate symptoms and improve the substantial skin-related morbidity and mortality experienced by this cohort.
